# [2-Oxido-1-naphthaldehyde (2-hydroxy­benzo­yl)hydrazonato]diphenyl­tin(IV)

**DOI:** 10.1107/S1600536809043591

**Published:** 2009-10-28

**Authors:** Jing Li, Handong Yin, Liyuan Wen, Jichun Cui

**Affiliations:** a College of Chemistry and Chemical Engineering, Liaocheng University, Shandong 252059, People’s Republic of China

## Abstract

In the title compound, [Sn(C_6_H_5_)_2_(C_18_H_12_N_2_O_3_)], the Sn^IV^ atom has a distorted trigonal-bipyramidal geometry. The Schiff base mol­ecule is coordinated to the Sn^IV^ atom in a tridentate fashion *via* the azomethine N atom, the hydr­oxy O atom and the carbonyl O atom. The complex involves an intra­molecular O—H⋯N hydrogen bond.

## Related literature

For related structures, see: Chen *et al.* (2006 ▶); Yearwood *et al.* (2002 ▶). For covalent radii, see: Sanderson (1967 ▶).
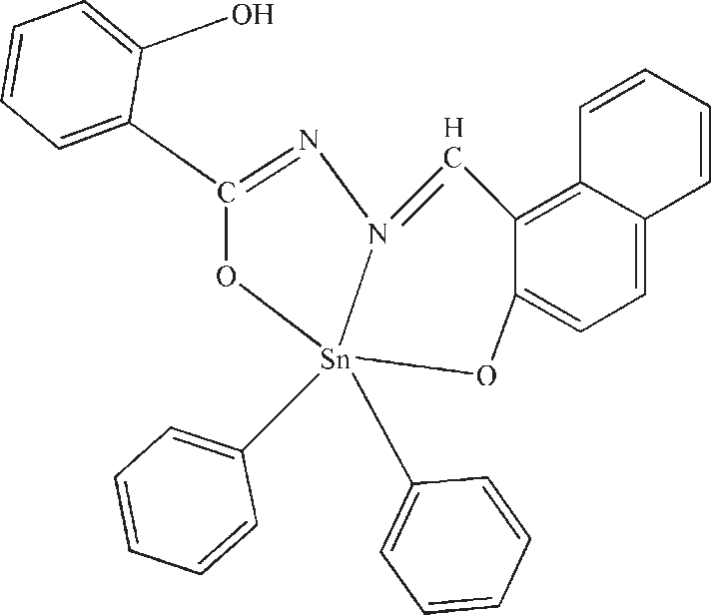


## Experimental

### 

#### Crystal data

[Sn(C_6_H_5_)_2_(C_18_H_12_N_2_O_3_)]
*M* *_r_* = 577.19
Monoclinic, 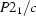

*a* = 9.418 (1) Å
*b* = 11.0861 (12) Å
*c* = 25.668 (2) Å
β = 109.547 (2)°
*V* = 2525.5 (4) Å^3^
*Z* = 4
Mo *K*α radiation
μ = 1.05 mm^−1^
*T* = 293 K
0.43 × 0.29 × 0.20 mm

#### Data collection

Siemens SMART 1000 CCD diffractometer
Absorption correction: multi-scan (*SADABS*; Sheldrick, 1996 ▶) *T*_min_ = 0.662, *T*_max_ = 0.818
12414 measured reflections
4435 independent reflections
3263 reflections with *I* > 2σ(*I*)
*R*_int_ = 0.031

#### Refinement

*R*[*F* ^2^ > 2σ(*F* ^2^)] = 0.033
*wR*(*F* ^2^) = 0.073
*S* = 1.03
4435 reflections
325 parameters
H-atom parameters constrained
Δρ_max_ = 0.43 e Å^−3^
Δρ_min_ = −0.33 e Å^−3^

### 

Data collection: *SMART* (Siemens, 1996 ▶); cell refinement: *SAINT* (Siemens, 1996 ▶); data reduction: *SAINT*; program(s) used to solve structure: *SHELXS97* (Sheldrick, 2008 ▶); program(s) used to refine structure: *SHELXL97* (Sheldrick, 2008 ▶); molecular graphics: *SHELXTL* (Sheldrick, 2008 ▶); software used to prepare material for publication: *SHELXTL*.

## Supplementary Material

Crystal structure: contains datablocks I, global. DOI: 10.1107/S1600536809043591/hy2239sup1.cif

Structure factors: contains datablocks I. DOI: 10.1107/S1600536809043591/hy2239Isup2.hkl

Additional supplementary materials:  crystallographic information; 3D view; checkCIF report

## Figures and Tables

**Table 1:** Selected bond lengths (Å)

Sn1—O1	2.121 (2)
Sn1—O3	2.061 (2)
Sn1—N2	2.154 (3)
Sn1—C19	2.106 (4)
Sn1—C25	2.113 (4)

**Table 2:** Hydrogen-bond geometry (Å, °)

*D*—H⋯*A*	*D*—H	H⋯*A*	*D*⋯*A*	*D*—H⋯*A*
O2—H2⋯N1	0.82	1.89	2.611 (5)	146

**Fig. 1.**
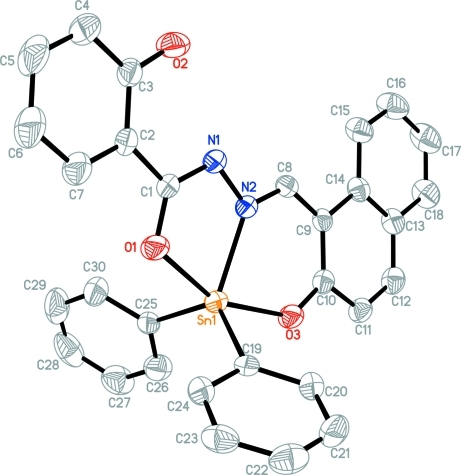
The molecular structure of the title compound, showing 50% probability displacement ellipsoids. H atoms have been omitted for clarity.

**Fig. 2.**
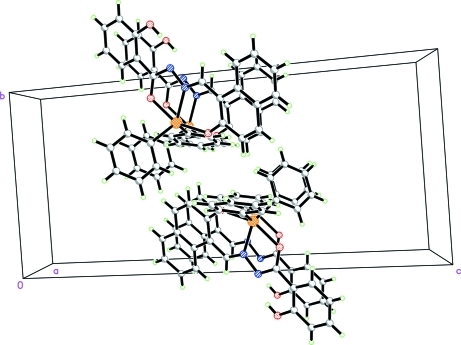
The crystal packing of the title compound.

## supplementary crystallographic information

### Comment

The molecular structure of the title compound is shown in Fig. 1. The Sn^IV^ atom is five-coordinated by two O atoms, two C atoms and one N atom. The distortion around the Sn^IV^ atom is a result of the constraints imposed by the Sn1–N2–N1–C1–O1 and Sn1–N2–C8–C9–C10–O3 rings. The dihedral angles between the two benzene rings (C19 to C24 and C25 to C30) and the O3–Sn1–N2 plane are 61.5 (1) and 67.2 (1)°, respectively. The Sn1—N2 distance is 2.154 (3) Å, close to the sum of the covalent radii (2.15 Å; Sanderson, 1967), indicating a strong Sn—N interaction. The O atoms coordinate to the Sn atom with one shorter and one longer Sn—O bond. Very similar structural parameters were observed in the compound studied by Yearwood *et al.* (2002). The angles at Sn1 confirm that the complex has a distorted trigonal-bipyramidal geometry.

### Experimental

2-Hydroxybenzhydrazide (5 mol) was added to 30 ml ethanol. The mixture was stirred for 0.5 h and then 2-hyroxy-1-naphthyldehyde (5 mol) was added, generating a yellow sediment immediately. The product was recrystallized from ethanol and DMF mixed solvent to get yellow crystals of 2-hydroxy-1-naphthaldehyde 2-benzoylhydrazone (*L*). The preparation of the title compound was carried out under nitrogen atmosphere. *L* (4 mmol) was added to a mixture of ethanol and benzene (v/v 1:3, 30 ml) with sodium ethoxide (4 mmol). The mixture was stirred for 0.5 h and then dichlorodiphenyltin (4 mmol) was added. The mixture was stirred for 12 h under reflux. After cooling to room temperature, the mixture was filtered and evaporated to dryness. The resulting solid was then recrystallized from dichloromethane-hexane (v/v 1:1). Analysis, calculated for C_30_H_22_N_2_O_3_Sn: C 62.42, H 3.84, N 4.85, O 8.32%; found: C 62.30, H 3.75, N 4.92, O 8.28%.

### Refinement

H atoms were positioned geometrically and refined as riding atoms, with C—H = 0.93 and O—H = 0.82 Å and with *U*_iso_(H) = 1.2(1.5 for hydroxyl)*U*_eq_(C, O).

### Crystal data

**Table:**

[Sn(C_6_H_5_)_2_(C_18_H_12_N_2_O_3_)]	*F*(000) = 1160
*M**_r_* = 577.19	*D*_x_ = 1.518 Mg m^−^^3^
Monoclinic, *P*2_1_/*c*	Mo *K*α radiation, λ = 0.71073 Å
Hall symbol: -P 2ybc	Cell parameters from 4435 reflections
*a* = 9.418 (1) Å	θ = 2.5–24.5°
*b* = 11.0861 (12) Å	µ = 1.05 mm^−^^1^
*c* = 25.668 (2) Å	*T* = 293 K
β = 109.547 (2)°	Block, colorless
*V* = 2525.5 (4) Å^3^	0.43 × 0.29 × 0.20 mm
*Z* = 4	

### Data collection

**Table:**

Siemens SMART 1000 CCD diffractometer	4435 independent reflections
Radiation source: fine-focus sealed tube	3263 reflections with *I* > 2σ(*I*)
graphite	*R*_int_ = 0.031
φ and ω scans	θ_max_ = 25.0°, θ_min_ = 1.7°
Absorption correction: multi-scan (*SADABS*; Sheldrick, 1996)	*h* = −11→11
*T*_min_ = 0.662, *T*_max_ = 0.818	*k* = −11→13
12414 measured reflections	*l* = −30→30

### Refinement

**Table:**

Refinement on *F*^2^	Primary atom site location: structure-invariant direct methods
Least-squares matrix: full	Secondary atom site location: difference Fourier map
*R*[*F*^2^ > 2σ(*F*^2^)] = 0.033	Hydrogen site location: inferred from neighbouring sites
*wR*(*F*^2^) = 0.073	H-atom parameters constrained
*S* = 1.03	*w* = 1/[σ^2^(*F*_o_^2^) + (0.0238*P*)^2^ + 1.7663*P*] where *P* = (*F*_o_^2^ + 2*F*_c_^2^)/3
4435 reflections	(Δ/σ)_max_ = 0.003
325 parameters	Δρ_max_ = 0.43 e Å^−^^3^
0 restraints	Δρ_min_ = −0.33 e Å^−^^3^

### Fractional atomic coordinates and isotropic or equivalent isotropic displacement parameters (Å^2^)

**Table:**

	*x*	*y*	*z*	*U*_iso_*/*U*_eq_	
Sn1	0.89811 (3)	0.74107 (2)	0.920886 (10)	0.04851 (10)	
N1	0.8396 (4)	0.4718 (2)	0.91811 (11)	0.0465 (8)	
N2	0.8343 (3)	0.5736 (2)	0.94938 (11)	0.0434 (7)	
O1	0.9135 (3)	0.6067 (2)	0.86422 (10)	0.0647 (8)	
O2	0.7809 (4)	0.2520 (2)	0.88057 (12)	0.0834 (10)	
H2	0.7848	0.3089	0.9015	0.125*	
O3	0.8815 (3)	0.8048 (2)	0.99407 (10)	0.0589 (7)	
C1	0.8817 (4)	0.4984 (3)	0.87560 (14)	0.0459 (9)	
C2	0.8935 (4)	0.4009 (3)	0.83892 (14)	0.0493 (9)	
C3	0.8422 (5)	0.2849 (3)	0.84222 (15)	0.0576 (11)	
C4	0.8529 (6)	0.1978 (4)	0.80504 (18)	0.0794 (14)	
H4	0.8193	0.1198	0.8075	0.095*	
C5	0.9126 (7)	0.2261 (5)	0.7647 (2)	0.0918 (17)	
H5	0.9166	0.1676	0.7392	0.110*	
C6	0.9665 (7)	0.3391 (5)	0.7614 (2)	0.1001 (19)	
H6	1.0100	0.3573	0.7347	0.120*	
C7	0.9554 (6)	0.4251 (4)	0.79798 (17)	0.0753 (14)	
H7	0.9905	0.5025	0.7954	0.090*	
C8	0.7840 (4)	0.5562 (3)	0.99031 (14)	0.0449 (9)	
H8	0.7550	0.4779	0.9950	0.054*	
C9	0.7683 (4)	0.6439 (3)	1.02883 (13)	0.0428 (9)	
C10	0.8202 (4)	0.7625 (3)	1.02946 (14)	0.0482 (9)	
C11	0.8110 (5)	0.8440 (3)	1.07056 (15)	0.0557 (10)	
H11	0.8460	0.9225	1.0708	0.067*	
C12	0.7523 (5)	0.8097 (4)	1.10938 (16)	0.0609 (11)	
H12	0.7496	0.8648	1.1364	0.073*	
C13	0.6946 (5)	0.6924 (4)	1.11023 (16)	0.0561 (11)	
C14	0.7010 (4)	0.6071 (3)	1.07003 (15)	0.0507 (10)	
C15	0.6398 (5)	0.4933 (4)	1.07211 (17)	0.0640 (12)	
H15	0.6401	0.4363	1.0455	0.077*	
C16	0.5797 (6)	0.4627 (4)	1.1119 (2)	0.0826 (15)	
H16	0.5410	0.3856	1.1122	0.099*	
C17	0.5756 (6)	0.5453 (5)	1.1519 (2)	0.0878 (16)	
H17	0.5346	0.5242	1.1790	0.105*	
C18	0.6327 (6)	0.6582 (4)	1.15106 (18)	0.0751 (14)	
H18	0.6308	0.7137	1.1780	0.090*	
C19	1.1244 (4)	0.7942 (3)	0.93967 (14)	0.0444 (9)	
C20	1.2167 (5)	0.8207 (3)	0.99286 (15)	0.0572 (11)	
H20	1.1779	0.8179	1.0217	0.069*	
C21	1.3657 (5)	0.8514 (4)	1.0035 (2)	0.0748 (13)	
H21	1.4275	0.8661	1.0396	0.090*	
C22	1.4234 (6)	0.8602 (4)	0.9612 (2)	0.0763 (13)	
H22	1.5236	0.8821	0.9686	0.092*	
C23	1.3339 (6)	0.8371 (4)	0.9084 (2)	0.0707 (13)	
H23	1.3723	0.8445	0.8796	0.085*	
C24	1.1862 (5)	0.8026 (3)	0.89768 (16)	0.0585 (11)	
H24	1.1268	0.7846	0.8616	0.070*	
C25	0.7141 (4)	0.8331 (3)	0.86445 (15)	0.0508 (10)	
C26	0.6817 (5)	0.9491 (4)	0.87485 (19)	0.0700 (12)	
H26	0.7412	0.9868	0.9071	0.084*	
C27	0.5624 (6)	1.0113 (5)	0.8384 (2)	0.0913 (16)	
H27	0.5436	1.0907	0.8459	0.110*	
C28	0.4723 (6)	0.9565 (7)	0.7916 (2)	0.0969 (19)	
H28	0.3911	0.9980	0.7674	0.116*	
C29	0.5006 (6)	0.8424 (7)	0.7803 (2)	0.0976 (18)	
H29	0.4386	0.8050	0.7484	0.117*	
C30	0.6228 (5)	0.7797 (4)	0.81640 (18)	0.0751 (13)	
H30	0.6427	0.7013	0.8079	0.090*	

### Atomic displacement parameters (Å^2^)

**Table:**

	*U*^11^	*U*^22^	*U*^33^	*U*^12^	*U*^13^	*U*^23^
Sn1	0.05637 (18)	0.04439 (15)	0.04495 (15)	−0.01070 (14)	0.01717 (12)	−0.00358 (12)
N1	0.058 (2)	0.0411 (16)	0.0412 (17)	−0.0028 (14)	0.0175 (16)	−0.0055 (13)
N2	0.048 (2)	0.0421 (16)	0.0400 (17)	−0.0043 (14)	0.0142 (15)	−0.0034 (13)
O1	0.094 (2)	0.0545 (16)	0.0572 (17)	−0.0219 (15)	0.0407 (17)	−0.0132 (13)
O2	0.138 (3)	0.0483 (16)	0.079 (2)	−0.0149 (18)	0.057 (2)	−0.0112 (15)
O3	0.080 (2)	0.0532 (15)	0.0504 (16)	−0.0166 (14)	0.0309 (15)	−0.0128 (12)
C1	0.048 (3)	0.046 (2)	0.039 (2)	−0.0072 (18)	0.0094 (18)	−0.0046 (16)
C2	0.054 (3)	0.054 (2)	0.036 (2)	−0.0022 (19)	0.0113 (19)	−0.0059 (17)
C3	0.071 (3)	0.053 (2)	0.045 (2)	0.008 (2)	0.014 (2)	−0.0043 (18)
C4	0.114 (4)	0.057 (3)	0.067 (3)	0.009 (3)	0.029 (3)	−0.016 (2)
C5	0.120 (5)	0.091 (4)	0.065 (3)	0.017 (3)	0.032 (3)	−0.029 (3)
C6	0.142 (6)	0.111 (4)	0.064 (3)	−0.014 (4)	0.056 (4)	−0.025 (3)
C7	0.097 (4)	0.079 (3)	0.057 (3)	−0.018 (3)	0.037 (3)	−0.015 (2)
C8	0.051 (3)	0.041 (2)	0.044 (2)	0.0003 (17)	0.0162 (19)	0.0026 (16)
C9	0.043 (2)	0.045 (2)	0.037 (2)	0.0062 (17)	0.0096 (17)	0.0028 (15)
C10	0.047 (2)	0.050 (2)	0.044 (2)	0.0035 (19)	0.0112 (17)	−0.0021 (18)
C11	0.062 (3)	0.050 (2)	0.052 (2)	0.002 (2)	0.016 (2)	−0.0086 (18)
C12	0.068 (3)	0.063 (3)	0.053 (3)	0.011 (2)	0.021 (2)	−0.010 (2)
C13	0.060 (3)	0.059 (2)	0.052 (2)	0.013 (2)	0.023 (2)	0.0021 (19)
C14	0.051 (3)	0.052 (2)	0.050 (2)	0.0134 (19)	0.018 (2)	0.0057 (18)
C15	0.082 (4)	0.058 (3)	0.066 (3)	0.005 (2)	0.044 (3)	0.003 (2)
C16	0.112 (5)	0.071 (3)	0.091 (4)	0.001 (3)	0.069 (3)	0.007 (3)
C17	0.118 (5)	0.089 (4)	0.084 (4)	0.014 (3)	0.071 (4)	0.014 (3)
C18	0.097 (4)	0.077 (3)	0.068 (3)	0.016 (3)	0.050 (3)	−0.003 (2)
C19	0.052 (2)	0.0325 (18)	0.047 (2)	−0.0046 (16)	0.0141 (19)	0.0030 (15)
C20	0.063 (3)	0.062 (3)	0.044 (2)	−0.007 (2)	0.014 (2)	0.0018 (18)
C21	0.059 (3)	0.078 (3)	0.068 (3)	−0.011 (3)	−0.004 (3)	−0.002 (2)
C22	0.055 (3)	0.067 (3)	0.106 (4)	−0.008 (2)	0.027 (3)	−0.003 (3)
C23	0.076 (4)	0.063 (3)	0.089 (4)	−0.010 (2)	0.048 (3)	0.002 (2)
C24	0.067 (3)	0.063 (2)	0.047 (2)	−0.010 (2)	0.022 (2)	−0.0004 (19)
C25	0.050 (3)	0.056 (2)	0.045 (2)	−0.0142 (19)	0.0134 (19)	0.0039 (17)
C26	0.059 (3)	0.066 (3)	0.077 (3)	−0.002 (2)	0.012 (3)	0.005 (2)
C27	0.080 (4)	0.081 (4)	0.106 (5)	0.008 (3)	0.021 (4)	0.023 (3)
C28	0.058 (4)	0.141 (6)	0.088 (4)	0.008 (4)	0.019 (3)	0.052 (4)
C29	0.070 (4)	0.153 (6)	0.055 (3)	−0.024 (4)	0.001 (3)	0.007 (4)
C30	0.076 (3)	0.087 (3)	0.054 (3)	−0.019 (3)	0.011 (2)	−0.006 (2)

### Geometric parameters (Å, °)

**Table:**

Sn1—O1	2.121 (2)	C13—C18	1.411 (5)
Sn1—O3	2.061 (2)	C13—C14	1.415 (5)
Sn1—N2	2.154 (3)	C14—C15	1.396 (5)
Sn1—C19	2.106 (4)	C15—C16	1.366 (5)
Sn1—C25	2.113 (4)	C15—H15	0.9300
N1—C1	1.313 (4)	C16—C17	1.387 (6)
N1—N2	1.395 (4)	C16—H16	0.9300
N2—C8	1.303 (4)	C17—C18	1.365 (6)
O1—C1	1.294 (4)	C17—H17	0.9300
O2—C3	1.348 (5)	C18—H18	0.9300
O2—H2	0.8200	C19—C20	1.383 (5)
O3—C10	1.315 (4)	C19—C24	1.389 (5)
C1—C2	1.462 (5)	C20—C21	1.380 (6)
C2—C3	1.386 (5)	C20—H20	0.9300
C2—C7	1.388 (5)	C21—C22	1.370 (6)
C3—C4	1.384 (5)	C21—H21	0.9300
C4—C5	1.371 (6)	C22—C23	1.359 (6)
C4—H4	0.9300	C22—H22	0.9300
C5—C6	1.364 (7)	C23—C24	1.378 (6)
C5—H5	0.9300	C23—H23	0.9300
C6—C7	1.366 (6)	C24—H24	0.9300
C6—H6	0.9300	C25—C26	1.368 (5)
C7—H7	0.9300	C25—C30	1.379 (5)
C8—C9	1.429 (5)	C26—C27	1.382 (6)
C8—H8	0.9300	C26—H26	0.9300
C9—C10	1.401 (5)	C27—C28	1.360 (7)
C9—C14	1.461 (5)	C27—H27	0.9300
C10—C11	1.414 (5)	C28—C29	1.344 (8)
C11—C12	1.346 (5)	C28—H28	0.9300
C11—H11	0.9300	C29—C30	1.397 (7)
C12—C13	1.413 (6)	C29—H29	0.9300
C12—H12	0.9300	C30—H30	0.9300
			
O3—Sn1—C19	94.31 (12)	C18—C13—C14	119.4 (4)
O3—Sn1—C25	99.45 (13)	C12—C13—C14	119.6 (3)
C19—Sn1—C25	123.88 (13)	C15—C14—C13	117.2 (3)
O3—Sn1—O1	155.42 (10)	C15—C14—C9	124.3 (3)
C19—Sn1—O1	93.27 (12)	C13—C14—C9	118.4 (3)
C25—Sn1—O1	95.61 (13)	C16—C15—C14	122.2 (4)
O3—Sn1—N2	82.61 (10)	C16—C15—H15	118.9
C19—Sn1—N2	122.65 (12)	C14—C15—H15	118.9
C25—Sn1—N2	113.00 (13)	C15—C16—C17	120.7 (4)
O1—Sn1—N2	73.63 (10)	C15—C16—H16	119.6
C1—N1—N2	112.1 (3)	C17—C16—H16	119.6
C8—N2—N1	115.9 (3)	C18—C17—C16	119.0 (4)
C8—N2—Sn1	128.2 (2)	C18—C17—H17	120.5
N1—N2—Sn1	115.7 (2)	C16—C17—H17	120.5
C1—O1—Sn1	115.0 (2)	C17—C18—C13	121.4 (4)
C3—O2—H2	109.5	C17—C18—H18	119.3
C10—O3—Sn1	133.8 (2)	C13—C18—H18	119.3
O1—C1—N1	123.6 (3)	C20—C19—C24	117.7 (4)
O1—C1—C2	117.9 (3)	C20—C19—Sn1	122.6 (3)
N1—C1—C2	118.6 (3)	C24—C19—Sn1	119.7 (3)
C3—C2—C7	118.0 (3)	C21—C20—C19	120.6 (4)
C3—C2—C1	122.9 (3)	C21—C20—H20	119.7
C7—C2—C1	119.1 (4)	C19—C20—H20	119.7
O2—C3—C4	117.6 (4)	C22—C21—C20	120.5 (4)
O2—C3—C2	122.6 (3)	C22—C21—H21	119.8
C4—C3—C2	119.9 (4)	C20—C21—H21	119.8
C5—C4—C3	120.2 (5)	C23—C22—C21	119.9 (5)
C5—C4—H4	119.9	C23—C22—H22	120.1
C3—C4—H4	119.9	C21—C22—H22	120.1
C6—C5—C4	120.8 (4)	C22—C23—C24	120.0 (4)
C6—C5—H5	119.6	C22—C23—H23	120.0
C4—C5—H5	119.6	C24—C23—H23	120.0
C5—C6—C7	118.9 (5)	C23—C24—C19	121.3 (4)
C5—C6—H6	120.5	C23—C24—H24	119.3
C7—C6—H6	120.5	C19—C24—H24	119.3
C6—C7—C2	122.2 (5)	C26—C25—C30	117.7 (4)
C6—C7—H7	118.9	C26—C25—Sn1	120.3 (3)
C2—C7—H7	118.9	C30—C25—Sn1	121.9 (3)
N2—C8—C9	127.4 (3)	C25—C26—C27	121.4 (5)
N2—C8—H8	116.3	C25—C26—H26	119.3
C9—C8—H8	116.3	C27—C26—H26	119.3
C10—C9—C8	122.0 (3)	C28—C27—C26	120.0 (5)
C10—C9—C14	119.2 (3)	C28—C27—H27	120.0
C8—C9—C14	118.8 (3)	C26—C27—H27	120.0
O3—C10—C9	124.0 (3)	C29—C28—C27	120.1 (5)
O3—C10—C11	115.9 (3)	C29—C28—H28	120.0
C9—C10—C11	120.0 (3)	C27—C28—H28	120.0
C12—C11—C10	121.0 (4)	C28—C29—C30	120.3 (5)
C12—C11—H11	119.5	C28—C29—H29	119.9
C10—C11—H11	119.5	C30—C29—H29	119.9
C11—C12—C13	121.8 (4)	C25—C30—C29	120.5 (5)
C11—C12—H12	119.1	C25—C30—H30	119.7
C13—C12—H12	119.1	C29—C30—H30	119.7
C18—C13—C12	121.0 (4)		
			
C1—N1—N2—C8	175.7 (3)	C11—C12—C13—C18	−179.6 (4)
C1—N1—N2—Sn1	−0.9 (4)	C11—C12—C13—C14	1.3 (6)
O3—Sn1—N2—C8	11.5 (3)	C18—C13—C14—C15	2.1 (6)
C19—Sn1—N2—C8	101.9 (3)	C12—C13—C14—C15	−178.8 (4)
C25—Sn1—N2—C8	−85.7 (3)	C18—C13—C14—C9	−178.8 (4)
O1—Sn1—N2—C8	−174.9 (3)	C12—C13—C14—C9	0.3 (6)
O3—Sn1—N2—N1	−172.4 (2)	C10—C9—C14—C15	177.3 (4)
C19—Sn1—N2—N1	−82.1 (3)	C8—C9—C14—C15	−4.9 (6)
C25—Sn1—N2—N1	90.4 (3)	C10—C9—C14—C13	−1.8 (5)
O1—Sn1—N2—N1	1.2 (2)	C8—C9—C14—C13	176.0 (3)
O3—Sn1—O1—C1	14.0 (5)	C13—C14—C15—C16	−1.8 (7)
C19—Sn1—O1—C1	121.8 (3)	C9—C14—C15—C16	179.2 (4)
C25—Sn1—O1—C1	−113.7 (3)	C14—C15—C16—C17	0.7 (8)
N2—Sn1—O1—C1	−1.3 (3)	C15—C16—C17—C18	0.0 (8)
C19—Sn1—O3—C10	−137.9 (3)	C16—C17—C18—C13	0.4 (8)
C25—Sn1—O3—C10	96.7 (3)	C12—C13—C18—C17	179.4 (5)
O1—Sn1—O3—C10	−30.3 (5)	C14—C13—C18—C17	−1.5 (7)
N2—Sn1—O3—C10	−15.5 (3)	O3—Sn1—C19—C20	17.9 (3)
Sn1—O1—C1—N1	1.4 (5)	C25—Sn1—C19—C20	122.4 (3)
Sn1—O1—C1—C2	−178.6 (3)	O1—Sn1—C19—C20	−138.7 (3)
N2—N1—C1—O1	−0.3 (5)	N2—Sn1—C19—C20	−66.0 (3)
N2—N1—C1—C2	179.7 (3)	O3—Sn1—C19—C24	−162.6 (3)
O1—C1—C2—C3	−170.6 (4)	C25—Sn1—C19—C24	−58.2 (3)
N1—C1—C2—C3	9.4 (6)	O1—Sn1—C19—C24	40.8 (3)
O1—C1—C2—C7	7.9 (6)	N2—Sn1—C19—C24	113.5 (3)
N1—C1—C2—C7	−172.1 (4)	C24—C19—C20—C21	−1.6 (6)
C7—C2—C3—O2	179.7 (4)	Sn1—C19—C20—C21	177.9 (3)
C1—C2—C3—O2	−1.8 (6)	C19—C20—C21—C22	2.3 (7)
C7—C2—C3—C4	−0.5 (6)	C20—C21—C22—C23	−0.9 (7)
C1—C2—C3—C4	178.0 (4)	C21—C22—C23—C24	−1.2 (7)
O2—C3—C4—C5	179.3 (5)	C22—C23—C24—C19	1.9 (7)
C2—C3—C4—C5	−0.6 (7)	C20—C19—C24—C23	−0.5 (6)
C3—C4—C5—C6	1.9 (8)	Sn1—C19—C24—C23	180.0 (3)
C4—C5—C6—C7	−2.1 (9)	O3—Sn1—C25—C26	37.8 (3)
C5—C6—C7—C2	1.1 (9)	C19—Sn1—C25—C26	−64.0 (4)
C3—C2—C7—C6	0.2 (7)	O1—Sn1—C25—C26	−161.7 (3)
C1—C2—C7—C6	−178.4 (5)	N2—Sn1—C25—C26	123.7 (3)
N1—N2—C8—C9	179.4 (3)	O3—Sn1—C25—C30	−142.1 (3)
Sn1—N2—C8—C9	−4.6 (6)	C19—Sn1—C25—C30	116.1 (3)
N2—C8—C9—C10	−5.8 (6)	O1—Sn1—C25—C30	18.4 (3)
N2—C8—C9—C14	176.5 (4)	N2—Sn1—C25—C30	−56.2 (4)
Sn1—O3—C10—C9	11.6 (6)	C30—C25—C26—C27	−0.4 (7)
Sn1—O3—C10—C11	−169.6 (3)	Sn1—C25—C26—C27	179.7 (4)
C8—C9—C10—O3	2.8 (6)	C25—C26—C27—C28	1.4 (8)
C14—C9—C10—O3	−179.5 (3)	C26—C27—C28—C29	−1.0 (8)
C8—C9—C10—C11	−176.0 (3)	C27—C28—C29—C30	−0.3 (8)
C14—C9—C10—C11	1.7 (5)	C26—C25—C30—C29	−0.9 (7)
O3—C10—C11—C12	−179.0 (4)	Sn1—C25—C30—C29	179.0 (4)
C9—C10—C11—C12	−0.2 (6)	C28—C29—C30—C25	1.2 (8)
C10—C11—C12—C13	−1.4 (6)		

### Hydrogen-bond geometry (Å, °)

**Table:**

*D*—H···*A*	*D*—H	H···*A*	*D*···*A*	*D*—H···*A*
O2—H2···N1	0.82	1.89	2.611 (5)	146
